# Influence of MXene (Ti_3_C_2_) Phase Addition on the Microstructure and Mechanical Properties of Silicon Nitride Ceramics

**DOI:** 10.3390/ma13225221

**Published:** 2020-11-19

**Authors:** Jaroslaw Wozniak, Mateusz Petrus, Tomasz Cygan, Artur Lachowski, Bogusława Adamczyk-Cieślak, Dorota Moszczyńska, Agnieszka Jastrzębska, Tomasz Wojciechowski, Wanda Ziemkowska, Andrzej Olszyna

**Affiliations:** 1 Faculty of Material Science and Engineering, Warsaw University of Technology, Wołoska 141 Str, 02-507 Warsaw, Poland; mateusz.petrus.dokt@pw.edu.pl (M.P.); tomasz.cygan.dokt@pw.edu.pl (T.C.); artur.lachowski.dokt@pw.edu.pl (A.L.); boguslawa.cieslak@pw.edu.pl (B.A.-C.); dorota.moszczynska@pw.edu.pl (D.M.); agnieszka.jastrzebska@pw.edu.pl (A.J.); andrzej.olszyna@pw.edu.pl (A.O.); 2 Faculty of Chemistry, Warsaw University of Technology, Noakowskiego 3, 00-664 Warsaw, Poland; twojciechowski@ch.pw.edu.pl (T.W.); ziemk@ch.pw.edu.pl (W.Z.)

**Keywords:** sintering, composites, mechanical properties, Si_3_N_4_

## Abstract

This paper discusses the influence of Ti_3_C_2_ (MXene) addition on silicon nitride and its impact on the microstructure and mechanical properties of the latter. Composites were prepared through powder processing and sintered using the spark plasma sintering (SPS) technic. Relative density, hardness and fracture toughness, were analyzed. The highest fracture toughness at 5.3 MPa·m^1/2^ and the highest hardness at HV5 2217 were achieved for 0.7 and 2 wt.% Ti_3_C_2_, respectively. Moreover, the formation of the Si_2_N_2_O phase was observed as a result of both the MXene addition and the preservation of the α-Si_3_N_4_→β-Si_3_N_4_ phase transformation during the sintering process.

## 1. Introduction

Silicon nitride is used in many different applications thanks to its excellent mechanical properties at elevated temperatures, thermal shock resistance and good tribological and wear properties [1,2,3,4]. Silicon nitride crystallizes in two allotropic forms: low-temperature α-Si_3_N_4_ and high-temperature β-Si_3_N_4_. Both types have a hexagonal structure but different stacking sequences: ABCD in the case of α-Si_3_N_4_ and ABAB in β-Si_3_N_4_. This affects the properties of individual phases: α-Si_3_N_4_ is characterized by higher hardness, while β-Si_3_N_4_ has higher chemical resistance. The transformation of α into β takes place within the temperature range of 1300 to 1800 °C. The α-Si_3_N_4_→β-Si_3_N_4_ transformation requires the presence of a liquid phase and consists of dissolving α-Si_3_N_4_ in a liquid and precipitation on the β-Si_3_N_4_ nuclei. The grains of the β-Si_3_N_4_ phase have elongated shapes and their morphology resembles whiskers [5,6]. The α-Si_3_N_4_→β-Si_3_N_4_ transformation is a factor that facilitates densification during the sintering process. In addition to controlling the amount of the β-Si_3_N_4_ phase in the ceramic sinter and the texturization of the β-Si_3_N_4_, this transformation can be used to improve the mechanical properties of the produced Si_3_N_4_ ceramic [7]. The preparation of self-reinforced Si_3_N_4_ allows a high fracture toughness, i.e., greater than 11 MPa·m^1/2^, to be obtained; however, it has a negative impact on the bending strength [8,9]. A significant improvement of this parameter can be achieved by texturizing the Si_3_N_4_ microstructure [1]. Hirao et al. [9] produced textured β-Si_3_N_4_ ceramics with a fracture toughness of 11.1 MPa·m^1/2^ and a bending strength of 1.1 GPa. They used seeding and tape casting methods. Teshima el al. [10] obtained high values of fracture toughness (14 MPa·m^1/2^) and bending strength (1.4 GPa) for ceramics produced using seeding and extrusion. In addition to the above-mentioned methods, matrix composites can also be used to improve the i properties of Si_3_N_4_. Components such as TiN, SiC whiskers, ZrO_2_ nanofibers and graphene can be used as reinforcing phases [11,12,13,14,15,16]. Very interesting research results have been obtained in the case of composites reinforced with graphene. Cygan et al. [2] produced Si_3_N_4_-1 wt.% Gn composites using the spark plasma sintering (SPS) method. They determined the influence of the sintering temperature on the β-Si_3_N_4_ properties and phase content formed during the consolidation of the composites. They determined the optimal sintered temperature to be 1700 °C, and observed a stabilization of the α-Si_3_N_4_ phase through the addition of graphene. Similar results have been presented by Rutkowski et al. [16]. They observed a decrease in the β-Si_3_N_4_ phase content with an increase of the graphene amount for composites consolidated with an hot pressing (HP) technic. In addition to the studies listed above, MXene phases have recently attracted a lot of attention. MXene phases are a large family of materials (carbides and nitrides) with two-dimensional (2D) structures. The strong covalent bonds between M–X entail high mechanical properties, making these materials potentially useful as reinforcing phases for composites. Additionally, the good conductivity and excellent volumetric capacity of these materials mean that they are commonly used in many industries, such as medicine, optoelectronics and energy storage. MXene phases are synthesized from MAX phases. They constitute a large group of anisotropic crystalline materials and their name reflects their composition, i.e., M_n+1_AX_n_, where M is a light transition metal, e.g., Ti, Nb, V; A is a metal from group 13 or 14; and X is carbon or nitrogen. The MAX phases exhibit a layered structure in which there is a metallic layer between M–X. In the process of synthesizing the MXene phases, this metallic layer is etched and then the products are removed in the rinsing process. In the final stage, the etched structures are delaminated into 2D structures. With regard to MXene phases, one great difficulty in using them as reinforcing phases is their thermal stability [17,18,19]. Depending on the conditions, the decomposition of MXene can occur at temperatures as low as 200 °C in an air atmosphere. In argon, the thermal stability of the MXene phases is much higher. The formation of anatase has been observed at temperatures above 800 °C. In both cases, the MXene was completely oxidized above 1000 °C and rutile was the final oxidized product [20,21]. As of yet, there are no reports in the literature on the use of MXene as a reinforcing phase in Si_3_N_4_ matrix composites. Only the preparation processes of composites based on SiC- and Al_2_O_3_-reinforced MXene have been described [22,23]. In both studies, the authors concluded that the addition of MXene improved the mechanical properties of the produced ceramics.

The use of the SPS method is extremely important with regard to limiting the MXene oxidation. Due to short heating and cooling times and the potential complete elimination of dwell time, it is possible to reduce the MXene phase decomposition, as in the case of composites reinforced with graphene [24]. For ceramic composites reinforced with graphene, it has been confirmed that the graphitization of graphene particles was reduced by using the SPS method. Moreover, a short sintering time and a lower sintering temperature also affected the properties of ceramics by limiting grain growth [25].

The main aim of this paper is to determine the effect of the addition of MXene to Si_3_N_4_ on the microstructure and mechanical properties of the obtained ceramics. To the best of our knowledge, this is the first study on the sintering of Si_3_N_4_ with the addition of MXene.

## 2. Materials and Methods

In the experimental work carried out to determine the effect of the addition of MXene to Si_3_N_4_, the ceramics were produced using a powder metallurgy technique. α-Si_3_N_4_ (Grade M11, H.C. Starck, Goslar, Germany) with a chemical purity of 99.95% and average particle size of 0.6 μm was used as a starting powder. Two sintering additives—2 wt.% MgO (Inframat Advance Materials, Manchester, CT, USA) with a chemical purity of 99.9% and average particle size of 0.3 μm and 2 wt.% ZrO_2_ (Tosoh Corpotation, Tokyo, Japan) with a chemical purity of 99.8% and average particle size of 0.4 μm—were used to improve the consolidation of the obtained ceramics. A series of five ceramics with different additions of Ti_3_C_2_–MXene (0.2, 0.5, 0.7, 1, 1.5, 2, 2.5 and 3 wt.%) were produced. Additionally, a ceramic without Ti_3_C_2_, used as a reference sample, and Si_3_N_4_-3 wt.% Ti_3_C_2_ without sintering additives were produced. Ti_3_AlC_2_ (MAX phase) was used as a starting material for the preparation of the MXene. It was synthesized using the SPS technique. Powdered Ti, Al and C were mixed in a ratio of 3:1:1.9 in a ball mill for 24 h. After drying and granulation, they were subjected to SPS synthesis (SPS HP D10, FCT Systeme GmbH, Effelder-Rauenstein, Germany) at a temperature of 1300 °C for 3 min under a vacuum. The material prepared in this way was then grinded. The preparation of MXene Ti_3_C_2_ was carried out through the acidic etching of the MAX Ti_3_AlC_2_ phase with concentrated (48%) hydrofluoric (HF) acid, under a fume hood. The MAX phase was slowly added to the HF, in portions, in amounts of 1 g MAX per 10 g of HF. The mixture was then stirred at 1000 rpm for 24 h until all available Al layers were removed. The resulting precipitate was separated from the acidic mixture and washed thoroughly with distilled water until the pH of the clay reached c.a. 7. The obtained Ti_3_C_2_ MXene was washed with ethanol, allowed to dry for 24 h at room temperature (RT) and then stored at 5 °C in the dark.

The obtained MXene powder was then subjected to the second stage of processing, in which the lamellar structure of the MXene was fragmented into single, multilayer (ML) flakes using a stepwise sonication process. This process was recently developed by the authors [26] specifically to avoid delamination into single-layered flakes, which are known to rapidly oxidize [27] and decompose in the course of the sintering process. Recent theoretical and experimental studies have shown that ML flakes are more stable compared to single-layered flakes [28]. Briefly, the first step of the sonication process was carried out in dried nonpolar hexane in a ratio of 1 g of powder to 50 cm^3^ of solvent for 2 h. To avoid overheating, an ice bath was used, together with a periodic working mode (1 s working, 3 s resting). The sediment was collected and dried for 2 h at RT and then subjected to sonication in dried polar isopropanol for 1 h via the same sonication procedure as in the case of hexane. After drying at RT, the 2D ML-Ti_3_C_2_T_x_ flakes were stored at 5 °C in the dark.

The prepared powder mixtures were homogenized in a planetary mill for 10 h in an isopropyl alcohol suspension. As a grinding media, 2 mm diameter ZrO_2_ balls were used with a powder-to-ball ratio of 1:10. The powder mixtures were dried at 50 °C for 24 h and then granulated using a #325 mesh sieve. The prepared mixtures were then consolidated using the SPS method. The sintering parameters used were as follows: temperature 1750 °C, heating/cooling rate 300 °C/min, dwell time 30 min, applied pressure 30 MPa, vacuum atmosphere 5 × 10^−2^ mbar and graphitic die and lining.

The properties of the obtained ceramics were measured using an Ultrapycnometer 1000 helium pycnometer (Quantachrome Instruments, Graz, Austria) for the skeletal density and a Vickers Hardness Tester (FV-700e, Future Tech, Kawasaki-City, Japan) for the Vickers hardness and fracture toughness (indentation method) under a load of 49 N. Before the density measurement, the samples were dried for 48 h at 60 °C. Hardness measurements were taken in accordance with EN ISO 6507-1:2007. A fracture toughness assessment was undertaken through measurement of crack lengths, propagating from the corners of the indentation as a result of pressing the Vickers indenter into the composite surface. The Niihara, Morena and Hasselman formulae were used to calculate K_IC_ [29]. In order to determine the mechanical properties of the ceramics, tests were carried out on three samples of a given composition, with at least 10 measurements for each sample. Microstructure observations of the ceramics, as well as the powders, were carried out using a scanning electron microscope (SEM Hitachi 5500, Hitachi, Tokyo, Japan). A transmission electron microscopy (TEM, Thermo Fisher Scientific, Hillsboro, OR, USA) specimen was prepared by mechanical grinding and subsequent Ar ion polishing at 4 keV to obtain an electron-transparent material. Observations were carried out using a TECNAI G2 F20 S-TWIN microscope operating at 200 kV. A Fischione 3000 high-angle annular dark field (HAADF) detector in scanning transmission electron microscopy (STEM) mode was used to collect images. The qualitative and quantitative phase compositions were analyzed using X-ray diffraction (XRD) (Bruker D8 ADVANCE X-ray diffractometer, Bruker Corporation, Billerica, MA, USA) with radiation Cu Kα (λ = 0.154056 nm).

## 3. Results

**Figure 1:**
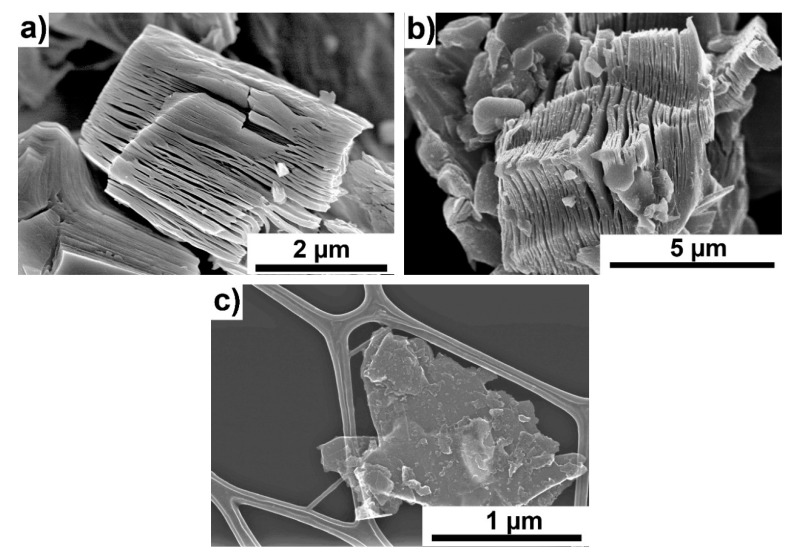
Morphology of Ti_3_AlC_2_ powder. (**a**) Ti_3_AlC_2_ MAX phases, (**b**) after hydrofluoric (HF) etching, (**c**) after delamination (Ti_3_C_2_ MXene).

**Figure 2:**
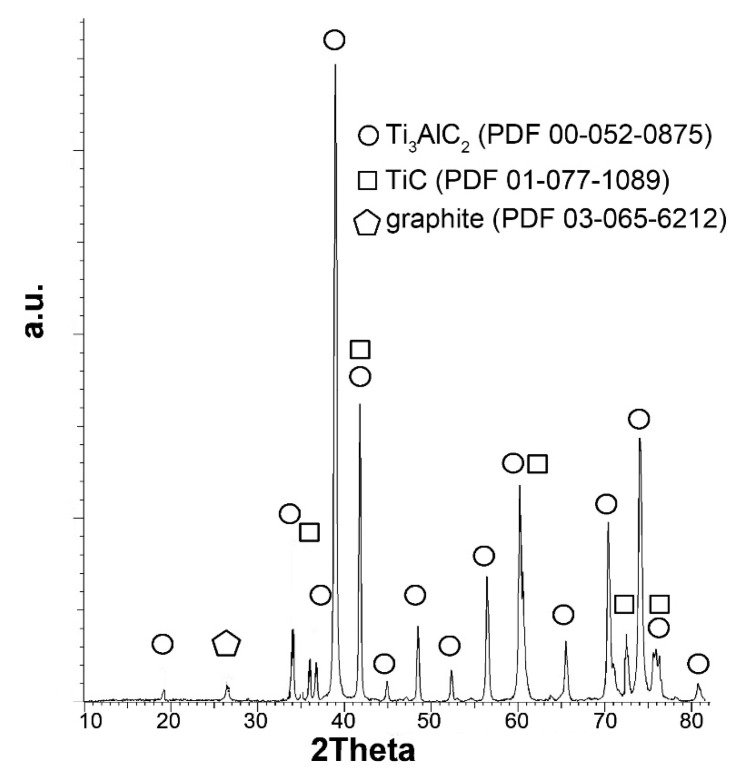
The X-ray diffraction (XRD) pattern obtained for synthesized Ti_3_AlC_2_.

Figure 1a,b show the morphology of the titanium carbide powder at each production stage. Figure 1a shows the Ti_3_AlC_2_ powder just after the synthesis and grinding process. The layered structure is visible; however, the individual layers are bonded by Al. The presence of the Ti_3_AlC_2_ phase was confirmed by the analysis of the phase composition, shown in Figure 2. It can be observed that the dominant component was Ti_3_AlC_2_; in addition to this phase, TiC was also formed as a result of the synthesis, and a small amount of unreacted graphite remained.

Figure 1b shows a powder particle after HF etching. It can be seen that the Ti_3_C_2_ layers are separated from each other. The morphology of MXene–Ti_3_C_2_ is shown in Figure 1c. The particles consisting of several layers of Ti_3_C_2_ are visible. The prepared powders were added to Si_3_N_4_ in order to determine their effect on the microstructure and properties of the obtained ceramics.

**Figure 3:**
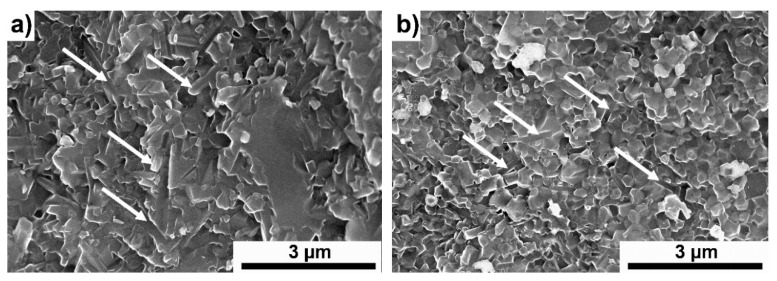
Fractures of (**a**) Si_3_N_4_ and (**b**) Si_3_N_4_ + 2 wt.% Ti_3_C_2_.

Figure 3a,b show the microstructures of a pure ceramic (Figure 3a) and of a specimen with the addition of 2 wt.% Ti_3_C_2_. In both cases, a fine-grained microstructure is visible. No large pores or discontinuities are visible. The presence of elongated grains of β-Si_3_N_4_ (marked with arrows in the figures), which were formed during the sintering process, can be observed. Moreover, it can be seen that in both the pure sample and the sample with the addition of MXene, transcrystalline fractures are the dominant fracture mechanism.

**Figure 4:**
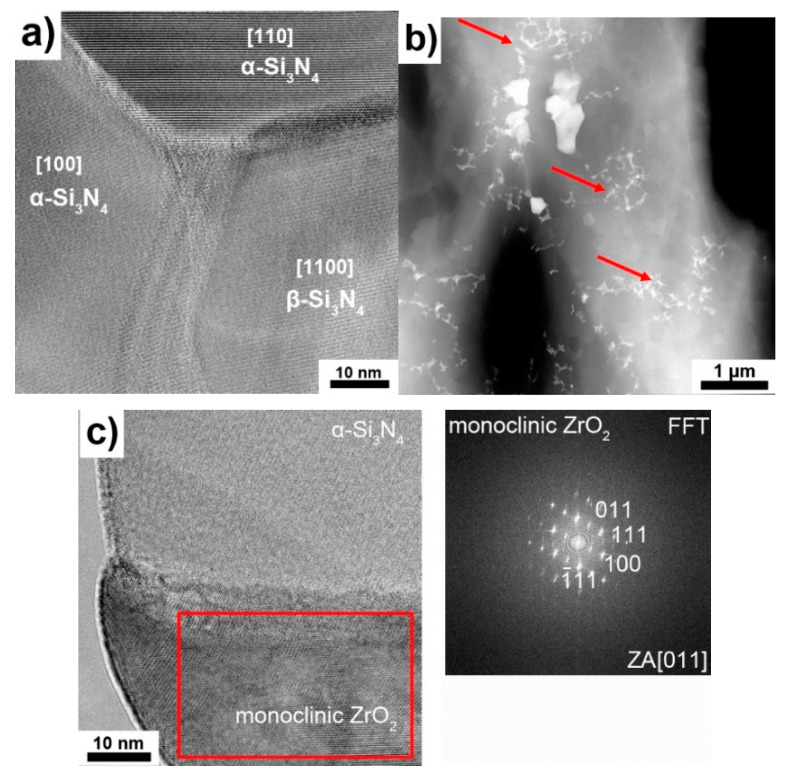
Transmission electron microscopy (TEM) analysis of Si_3_N_4_ + 2 wt.% Ti_3_C_2_. (**a**) Triple-point region of silicon nitride grains, (**b**) third phase located on Si_3_N_4_ grain boundaries, (**c**) high-resolution transmission electron microscopy (HRTEM) image and fast Fourier transform (FFT) pattern of third phase.

TEM analysis confirmed the observations from the scanning electron microscope. In the microstructures of the materials, both α-Si_3_N_4_ and β-Si_3_N_4_ phases with good cohesion between individual grains (Figure 4a) are visible. Also, no pores or discontinuities can be observed. Using STEM, a third phase located on the Si_3_N_4_ grain boundaries was revealed (indicated by arrows in Figure 4b). The HAADF detector shows mass thickness in contrast to higher signal intensity (brightness in the image), and so this must correspond to the presence of a relatively heavy element. In this case, the only possible element was zirconium. This was confirmed by high-resolution transmission electron microscopy (HRTEM) imaging (Figure 4c).

**Figure 5:**
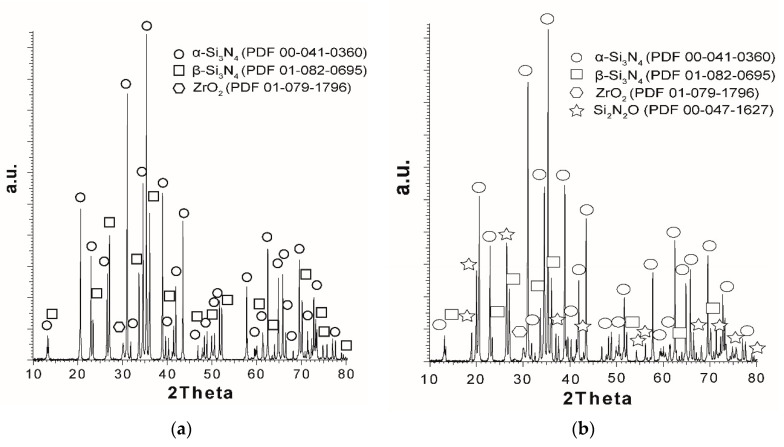

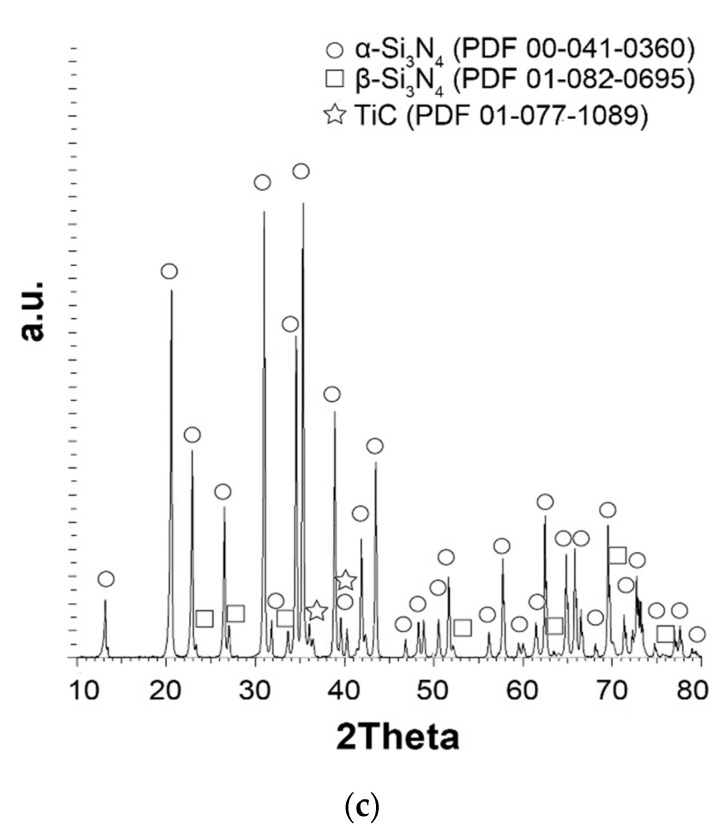
The XRD patterns of (**a**) Si_3_N_4_ ceramics, (**b**) Si_3_N_4_ + 3 wt.% Ti_3_C_2,_ (**c**) Si_3_N_4_ + 3 wt.% Ti_3_C_2_ (without sintering additives).

The fast Fourier transform (FFT) pattern obtained from the region marked with the red box in Figure 4c corresponds to a monoclinic ZrO_2_ phase. Again, no discontinuities in the Si_3_N_4_/ZrO_2_ grain boundaries can be seen. In order to determine the influence of Ti_3_C_2_ addition on the sintered silicon nitride, qualitative and semiquantitative phase analyses were performed. The results of the analyses are presented in Figure 5a–c and in Table 1. With regard to the data compiled in the table, only the main phases are presented. Hence, the sum of the phases does not equal 100%. Figure 5a shows the XRD pattern for the reference sample. It can be seen that during the sintering process, the α-Si_3_N_4_→β-Si_3_N_4_ phase transformation took place.

The amount of the β-Si_3_N_4_ phase is estimated at 27% (Table 1). The addition of MXene to the silicon nitride significantly increased the amount of the β-Si_3_N_4_ phase to 47% for 0.7 wt.% Ti_3_C_2_, and then decreased to 12 and 16% for 2 and 3 wt.% respectively. Moreover, in the case of the ceramics with the MXene addition, the appearance of a third Si_2_N_2_O phase can be observed (Figure 5b). The amount only slightly changed with the Ti_3_C_2_ content and was about 25% (Table 1).

The formation of the Si_2_N_2_O phase has already been described in the literature. Trigg et al. [30] proposed a reaction to form this phase in the presence of TiO_2_:

6TiO_2_ + 2Si_3_N_4_→6TiN + 6SiO_2_ + N_2_ (g) (1)

The formed SiO_2_ reacts with Si_3_N_4_ to form Si_2_N_2_O in the following reaction:

SiO_2_ + Si_3_N_4_→2Si_2_N_2_O (2)

In the case of our ceramics, TiO_2_ was not added during the powder mixing stage; however, it could form as a result of the oxidation of MXene during the sintering process. In the first stage of MXene phases oxidation, TiO_2_ was produced on their surfaces, leaving behind graphene-like carbon structures [21,31]. Their presence should have favored the formation of Si_2_N_2_O in accordance with reactions (1) and (2), but XRD phase analysis did not indicate the presence of TiN. However, the amount of this phase might have been below the detection threshold of the measurement method. Additionally, a ceramic using Ti_3_C_2_ without sintering activators was produced in order to determine whether Si_2_N_2_O phase formation is possible only in the presence of TiO_2_. On the basis of the obtained results (Figure 5c and Table 1), it can be seen that a very low level of β-Si_3_N_4_ content was present. This was due to the small amount of the liquid phase, which is necessary for the α-Si_3_N_4_→β-Si_3_N_4_ transformation. The small amount of this phase is related to the SiO_2_ which is present on the Si_3_N_4_ surface [5,6,32]. Moreover, the Si_2_N_2_O phase was not formed. This is the opposite of what is found in the literature. Wu et al. [33] investigated the effect of TiO_2_ and TiO_2_/ZrO_2_/Y_2_O_3_ addition to Si_3_N_4_ on the formation of the Si_2_N_2_O phase. They showed that Si_2_N_2_O does not form in ceramics with oxide sintering activators. In our case, the absence of the Si_2_N_2_O phase may be due to a small amount or lack of TiO_2_ in the structure. According to [34], a sintering process without an oxide agent may lead to changes in the oxidation process. Lotfi et al. showed that as a result of Ti_3_C_2_ annealing in a vacuum, Ti and C atoms were rearranged to form cubic titanium carbide (TiC). This confirms our results concerning the sintering of the sample without sintering additives. The XRD tests (Figure 5c) indicate the presence of TiC. The decrease in the β-Si_3_N_4_ phase content for samples with a higher amount of MXene is also very interesting. The blocking of the α-Si_3_N_4_→β-Si_3_N_4_ transformation can be caused by the presence of carbon structures formed at the initial stage of Ti_3_C_2_ oxidation. The range of the oxidation temperatures and α-Si_3_N_4_→β-Si_3_N_4_ transformations are similar. The presence of layered carbon structures may inhibit the growth of the β-Si_3_N_4_ phase. Similar results have been obtained for Si_3_N_4_ composites reinforced with graphene. The presence of carbon structures in the ceramics was not confirmed in our tests. However, to comply with the oxidation scheme of MXene available in the literature, it can be presumed that carbon structures were present in the microstructures within the range of the transformation temperature, and then completely decomposed at higher temperatures.

**Figure 6:**
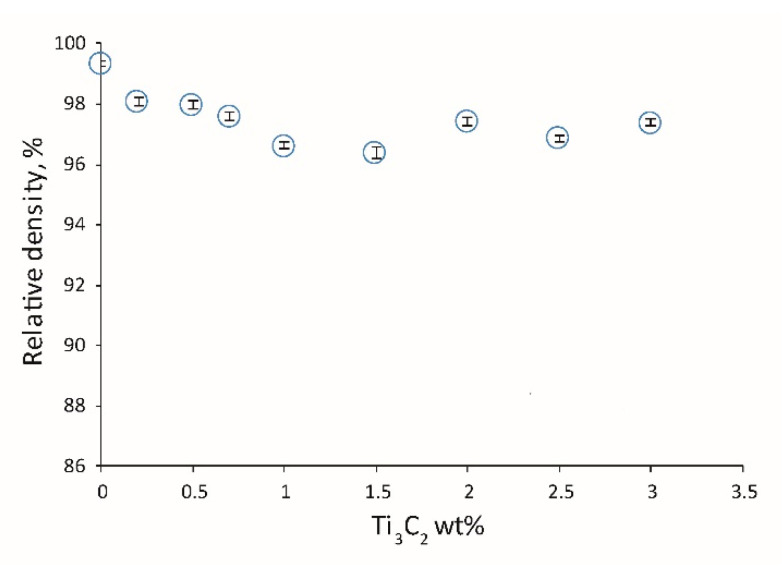
Influence of two-dimensional (2D) sheets of the Ti_3_C_2_ MXene weight content on the relative density of the composites.

In the next part of the research, the sample without sintering additives was omitted because the absence of such additives would result in a low degree of sample consolidation, making it impossible to perform mechanical tests for this sample. Figure 6 shows the density measurement results for the obtained ceramics. All samples with MXene addition demonstrated a lower density compared to the reference sample. The change in density as a function of the Ti_3_C_2_ phase content does not show any characteristic trend. The density results oscillate around 97%.

**Figure 7:**
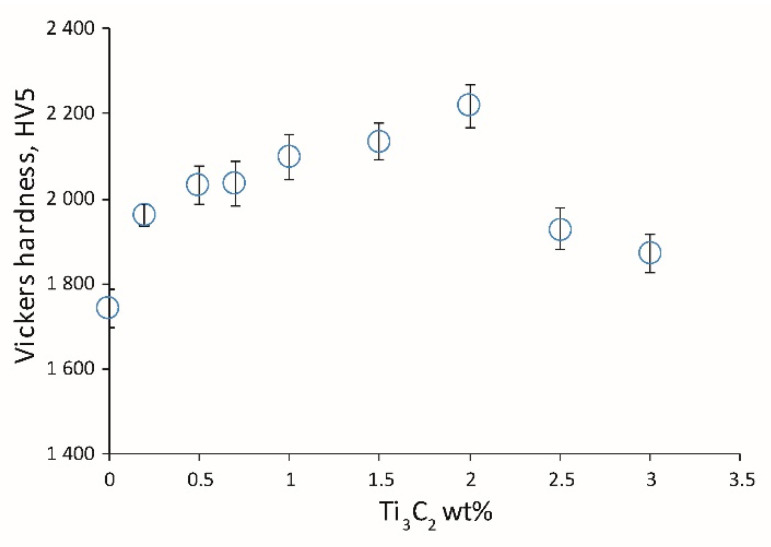
Influence of 2D sheets of the Ti_3_C_2_ MXene weight content on the Vickers hardness of the composites.

Figure 7 shows the influence of the Ti_3_C_2_ amount on the hardness of the obtained ceramics. An increase in hardness with increasing MXene content can be seen. The highest hardness was observed for the sample with 2 wt.% Ti_3_C_2_. After exceeding 2 wt.%, the hardness of the ceramic decreases. Based on the obtained results, it can be concluded that the addition of Ti_3_C_2_ also affects the mechanical properties of the ceramics. Despite the increase in the amount of the β-Si_3_N_4_ phase, in the case of lower MXene content, the hardness increases in comparison to the reference sample. Phase α-Si3N4 is hard compared to phase β-Si_3_N_4_ [1]. Moreover, it can be seen that there is an optimal content level for α-Si_3_N_4_ and β-Si_3_N_4_ phases for which the hardness has the highest value (the ceramic with the addition of 2 wt.% Ti_3_C_2_). The Si_2_N_2_O does not have a significant effect on the ceramics’ properties, since the mechanical properties of this phase are similar to those of Si_3_N_4_.

**Figure 8:**
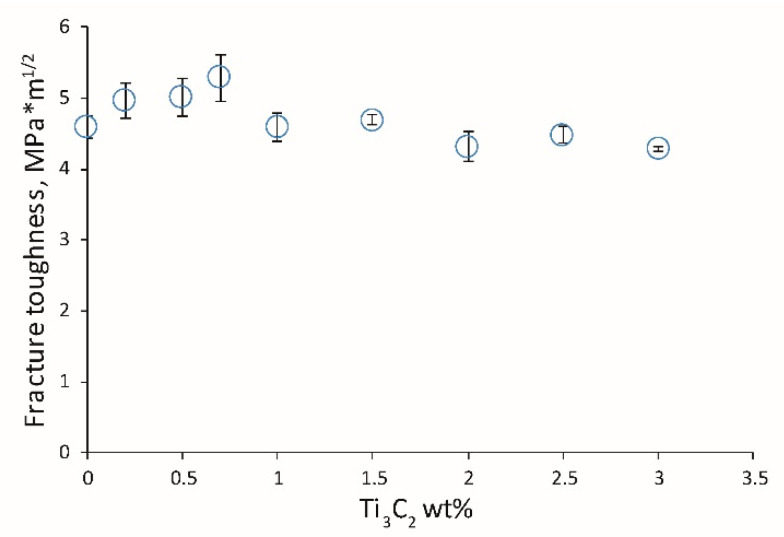
Influence of 2D sheets of the Ti_3_C_2_ MXene weight content on the fracture toughness of the composites.

Figure 8 shows the influence of MXene content on the fracture toughness of the obtained ceramics. There is an initial increase in K_IC_ to 0.7 wt.% Ti_3_C_2_, followed by a decrease. This kind of change in fracture toughness is related to the β-Si_3_N_4_ phase content. For higher amounts of β-Si_3_N_4_ phase, higher K_IC_ values are observed.

## 4. Conclusions

In this study, research was carried out to determine the effect of MXene phase addition on the mechanical properties and microstructure of Si_3_N_4_ ceramics. The obtained results indicate the absence of MXene phases after the sintering process. This proves that there is no possibility of producing MXene composites reinforced with the Si_3_N_4_ matrix with oxide sintering activators. The addition of Ti_3_C_2_, however, enables the modification of the phase composition of the ceramics by limiting the α-Si_3_N_4_→β-Si_3_N_4_ phase transformation and introducing an additional Si_2_N_2_O phase. By adding a certain amount of MXene, it is also possible to control mechanical properties such as hardness and fracture toughness.

## Figures and Tables

**Table 1:** Influence of Ti_3_C_2_ and sintering additive content on the α and β phase amounts in Si_3_N_4_ ceramics.

Sample ID	α-Si_3_N_4_ [%]	β-Si_3_N_4_ [%]	Si_2_N_2_O [%]
Si_3_N_4_	72	27	0
Si_3_N_4_ + 0.7 wt.% Ti_3_C_2_	30	47	22
Si_3_N_4_ + 2 wt.% Ti_3_C_2_	58	12	29
Si_3_N_4_ + 3 wt.% Ti_3_C_2_	59	16	24
Si_3_N_4_ + 3 wt.% Ti_3_C_2_ (without sintering additives)	94	4	0
